# Research Progress of AlGaN-Based Deep Ultraviolet Light-Emitting Diodes

**DOI:** 10.3390/mi14040844

**Published:** 2023-04-13

**Authors:** Ruiqiang Xu, Qiushi Kang, Youwei Zhang, Xiaoli Zhang, Zihui Zhang

**Affiliations:** 1 Guangdong Provincial Key Laboratory of Information Photonics Technology, School of Physics and Opto-Electronic Engineering, Guangdong University of Technology, Guangzhou 510006, China; 18475330011@163.com (R.X.); wzhgwb159@163.com (Y.Z.); 2 School of Integrated Circuits, Guangdong University of Technology, Guangzhou 510006, China; k13590049442@126.com (Q.K.);

**Keywords:** deep ultraviolet light-emitting diodes, internal quantum efficiency, light extraction efficiency, wall-plug efficiency

## Abstract

AlGaN-based deep ultraviolet light-emitting diodes (DUV LEDs) have great application prospects in sterilization, UV phototherapy, biological monitoring and other aspects. Due to their advantages of energy conservation, environmental protection and easy miniaturization realization, they have garnered much interest and been widely researched. However, compared with InGaN-based blue LEDs, the efficiency of AlGaN-based DUV LEDs is still very low. This paper first introduces the research background of DUV LEDs. Then, various methods to improve the efficiency of DUV LED devices are summarized from three aspects: internal quantum efficiency (IQE), light extraction efficiency (LEE) and wall-plug efficiency (WPE). Finally, the future development of efficient AlGaN-based DUV LEDs is proposed.

## 1. Introduction

As an important wide band gap semiconductor material, AlGaN is an ideal material for preparing deep ultraviolet light-emitting diodes (DUV LEDs) due to its excellent chemical stability and low electronic affinity. Over the past few decades, mercury lamps have been the most important deep ultraviolet (DUV) light source on the market. However, mercury is a toxic substance. Therefore, it has been of urgent interest to find an alternative DUV light source. Compared with the traditional mercury lamp DUV light source, AlGaN-based DUV LEDs have obvious advantages of energy conservation, environmental protection, easy miniaturization realization and broad application prospects in the fields of sterilization, biological monitoring, ultraviolet phototherapy, anticounterfeit detection, etc., especially in terms of sterilization and disinfection, against virus infestations such as COVID-19, for which the continuous improvement of applications meeting the demand for deep ultraviolet sterilization and disinfection market provides a golden opportunity for the development of DUV LEDs. However, at present, the external quantum efficiency (EQE) of most DUV LEDs is still less than 10%, resulting in their failure to effectively kill bacteria. Therefore, it is essential to improve the EQE of DUV LEDs. As we all know, the EQE is affected by internal quantum efficiency (IQE) and light extraction efficiency (LEE). Therefore, efforts must be contributed to improve the IQE and LEE of DUV LEDs. In this paper, we show the research background of DUV LEDs and review the methods used to enhance the efficiency of DUV LEDs in recent years. At the same time, we also discuss how to improve the wall-plug efficiency (WPE) of DUV LEDs.

In the 1990s, with the increasing efficiency of GaN-based blue or green light-emitting diodes and their gradual application in commercial production, many research groups conducted in-depth research on light-emitting diodes (LEDs) with smaller emission wavelengths. In the following ten years, the emission wavelengths of LEDs were extended to ultraviolet light. From 1996 to 1999, several research groups began to study AlGaN-based ultraviolet light-emitting diodes (UV LEDs) with wavelengths less than 360 nm. In 1999, Hirayama et al. reported the first efficient DUV (230 nm) photoluminescence (PL) from AlGaN/AlN quantum wells (QWs) [1] and a 330 nm band AlGaN-QW UV LED on SiC [2]. From 2002 to 2006, a research group at the University of South Carolina reported the first 250–280 nm AlGaN-based DUV LEDs [3,4,5]. In 2006, a group at NTT in Japan reported the shortest wavelength (210 nm) LED using an AlN-emitting layer [6]. With the deepening of research, many researchers have begun to focus on the innovative development of AlGaN-based DUV LEDs at wavelengths lower than 280 nm, because AlGaN-based DUV LEDs at such wavelengths are highly competitive and expected to have a huge market in the field of sterilization applications. In 2008, Hirayama et al. used thin quantum wells and AlN buffer layers to realize 227 nm AlGaN-based DUV LEDs with an output power of 0.15 mW [7]. In 2010, they reported 222 nm deep ultraviolet (DUV) AlGaN multiple quantum well (MQW) light-emitting diodes (LEDs) manufactured on high-quality AlN buffer layers grown on sapphire substrates [8]. In 2012, Kinoshita et al. fabricated AlGaN-based DUV LEDs on an AlN substrate with hydride vapor-phase epitaxy (HVPE), enhancing their output power [9].

In addition to the research conducted by many researchers on DUV LEDs, many companies also began to develop DUV LEDs for sterilization applications since 2010. In 2012, Sensor Electronic Technology (SET) reported a maximum EQE of 11% for 278 nm LEDs [10]. In 2013, Crystal IS and Tokuyama developed efficient 265 nm LEDs on bulk AlN substrates fabricated using a sublimation method [11,12] and hydride vapor-phase epitaxy (HVPE) [9,13], respectively. Nichia, Nikkiso and Nitek also developed efficient DUV LEDs.

Although all researchers have been working hard to develop and innovate AlGaN-based DUV LEDs, their current efficiency is still very low and cannot be well applied to commercial production on a large scale. For this reason, this paper gives the following three reasons for the inferiority of DUV LEDs:

1. The IQE of AlGaN-based DUV LEDs is relatively sensitive to the threading dislocation density (TDD), and the high level of TDD can seriously affect the device performance of DUV LEDs.
2. For AlGaN-based DUV LEDs, the hole concentration of the p-AlGaN layer and the electron injection efficiency are low.
3. The p-GaN layer in AlGaN-based DUV LEDs can absorb ultraviolet light, resulting in a low LEE.

Now, the IQE of the reported InGaN QWs has reached 80%. AlGaN QWs conventionally use high TDD AlN templates with IQEs as low as 1%. Therefore, in order to obtain an IQE of up to 80%, the TDD must be reduced to below 1 × 10^8^ cm^−2^. The use of AlN single-crystal wafers can effectively reduce the TDD and obtain a higher IQE, but they are expensive to use in commercial DUV LEDs. In addition, the device performance of AlGaN-based DUV LEDs strongly depends on the performance of the p-AlGaN. Due to its deep receptor level, the hole concentration of p-AlGaN with a high Al content (Al > 60%) is low (as low as 10^14^ cm^−3^) and the leakage of electrons into the p-side layer can lead to the reduction in the electron injection efficiency of DUV LEDs. Due to the lack of a high hole density in p-AlGaN, the p-GaN contact layer must be used. However, due to the strong absorption of DUV light in the p-GaN layer, the LEE must be significantly reduced. Generally, the LEE of fabricated DUV LEDs is less than 8%. Therefore, many researchers have proposed various methods to improve the LEE, such as using a transparent p-AlGaN layer and highly reflective p-type electrodes.

In general, parameters such as the WPE, EQE and light output power (LOP) are used to assess the photoelectric performance of DUV LEDs. The relationship between some main parameters can be expressed with the following formulas:

(1) $$\mathrm{WPE}=\frac{\mathrm{LOP}}{I\times V}=\mathrm{EQE}\times \mathrm{EE}$$

(2) $$\mathrm{EQE}=\frac{\mathrm{LOP}/\mathrm{hv}}{I/e}=\mathrm{IQE}\times \mathrm{LEE}=\mathrm{RRE}\times \mathrm{CIE}\times \mathrm{LEE}$$

where I and V are the operation current and voltage, respectively, h is the Planck constant, v is the frequency of the emitted light, e is the elementary charge, EE is the electrical efficiency, RRE is the radiative recombination efficiency and CIE is the carrier injection efficiency.

With regard to the efficiency of DUV LEDs, what follows describes the various methods found in recent years to be capable of improving the IQE from the aspect of crystalline quality, hole injection efficiency and electron injection efficiency, as well as some measures to enhance the LEE and WPE. By improving the IQE and LEE of DUV LEDs, the EQE and WPE are enhanced to improve the performance of the device.

## 2. Increase the IQE of DUV LEDs

It is well known that the IQE of DUV LEDs is affected by many factors, such as crystalline quality, electron injection efficiency and hole injection efficiency. Generally, the IQE of DUV LEDs can be expressed as the product of radiation recombination efficiency (RRE) and carrier injection efficiency (CIE); the RRE is the ratio of electron–hole pairs that participate in radiation recombination to the number of electron–hole pairs injected into the active region, while the CIE is the ratio of the number of electron–hole pairs injected into the active region to the number of electron–hole pairs injected into the LED device. In general, the ABC model [14] is often used to estimate the RRE using the following formula:

(3) $$\mathrm{RRE}=\frac{{\mathrm{Bn}}^{2}}{\mathrm{An}+{\mathrm{Bn}}^{2}+{\mathrm{Cn}}^{3}}$$

where the coefficients A, B and C represent the Shockley–Read–Hall (SRH) nonradiative recombination coefficient, radiative recombination coefficient and Auger recombination coefficient, respectively, and n is the carrier concentration. Generally speaking, the SRH process is related to defects in crystal materials. Defects in high Al component AlGaN films can form nonradiative recombination centers, resulting in LED devices having a higher TDD and lower RRE. In addition, an increase in the Auger recombination rate can reduce the RRE, possibly resulting in a gradual decrease in the light power of LEDs during operation due to hot carriers generated by the Auger recombination [15].

### 2.1. Improve the Crystalline Quality

Due to the development process of AlGaN-based DUV LEDs being similar to that of InGaN-based visible LEDs, it has been found that obtaining an epitaxial layer with a high crystalline quality is the prerequisite for manufacturing efficient DUV LEDs. It has also been found that DUV LED devices epitaxially grown on AlN homogeneous single-crystal substrates have excellent performance. Growth substrates have the advantages of a large band gap, good heat dissipation and low TDD, but they are expensive and small, which is not conducive to large-scale commercial production. Sapphire substrates have become the mainstream growth substrate of DUV LEDs due to their low price, good stability and simple preparation process. Generally, the AlN layer is used as a key template between a sapphire substrate and AlGaN material. However, a mismatch was found to exist between the AlN and sapphire in the lattice constant and thermal expansion coefficient [16,17], resulting in an AlN film with a very high TDD, usually between 10^9^ and 10^10^ cm^−2^. Due to the nonradiative recombination of carriers at the dislocation, a high TDD significantly reduces the IQE of multiple quantum wells (MQWs), which seriously affects the DUV LED devices’ performance. According to calculations [10], when the TDD is from 10^9^ to 10^10^ cm^−2^, the IQE of DUV LEDs can drop sharply, and a too high TDD can even cause the IQE to fall below 10% [10,18]. The intrinsic IQE can be measured experimentally using the low-temperature photoluminescence method, without involving carrier injection.

In view of the above situation, researchers have proposed many technologies to reduce the TDD of the AlN template, for example, the pulsed-flow multilayer AlN buffer growth technique [19] and migration-enhanced metal–organic chemical vapor deposition (MEMOCVD) technology [20,21]. Currently, the most commonly used technologies include the following: 1. The two-step growth method [22,23,24], which can alleviate the lattice mismatch between the AlN and sapphire substrate and improve the quality of AlN. Hasan et al. utilized a two-step process to grow a series of samples with thicknesses ranging from 1 to 4 μm, and reported 4 μm thick AlN layers with a total dislocation density of 1.1 × 10^9^ cm^−2^ [22]. However, the two-step growth method of the heteroepitaxial AlN on sapphire had certain limitations. Therefore, Zhang et al. introduced a three-step growth process, which further reduced the dislocation density by adding additional growth steps [24]. 2. The high-temperature annealing (HTA) of sputtered AlN on sapphire [25,26,27] was used by Miyake et al., who successfully grew a high-quality AlN layer on an annealed AlN buffer layer on a sapphire substrate, and achieved a TDD of 4.7 × 10^8^ cm^−2^ [25]. 3. The epitaxial lateral overgrowth (ELOG) method [28,29,30] used by Nakano et al., who for the first time demonstrated that ELOG AlN layers were grown on trench-patterned sapphire substrates, achieving a dislocation density of 6.7 × 10^8^ cm^−2^ [28]. In addition, ELOG techniques on microstripe-patterned sapphires or AlN/sapphire templates have received considerable attention, capable of significantly reducing the TDD and improving the IQE of DUV LEDs [31,32,33]. However, the large microstripe spacing on microstripe-patterned sapphires usually requires the coalescence thickness of AlN to be close to 10 μm. This means greatly increasing the growth time and cost. Therefore, in order to obtain a thinner coalescence thickness, nanopatterned sapphire substrates (NPSSs) were proposed and successfully fabricated. At present, researchers have studied the advantages of epitaxial growth on nanopatterned substrates in inhibiting the TDD and reducing the lifetime of the Shockley–Read–Hall (SRH) coefficient [34,35]. Compared with the growth of the Al-rich AlGaN layer on plane sapphire substrates, growth on nanopatterned sapphire substrates can obtain higher-quality AlGaN-based DUV LEDs. Of course, if the cost is not considered, the growth of Al-rich AlGaN layers on independent AlN substrates can significantly reduce the TDD, as low as 10^3^ to 10^5^ cm^−2^, thus, greatly improving the IQE of the DUV LEDs. Ref. [36] summarized the reported TDD values obtained through different growth technologies on different substrates. Combining the calculations, the intrinsic IQE of DUV LEDs can be estimated, thus, providing a clearer understanding of future technology research and development towards improving the intrinsic IQE.

### 2.2. Improve the Hole Injection Efficiency

As mentioned above, when the TDD is reduced to a certain level, the DUV LEDs can obtain a higher intrinsic IQE, but, in fact, the IQE is closely related to the carrier injection. Generally speaking, the hole injection layer of AlGaN-based DUV LEDs at present consists of a p-type electron barrier layer (p-EBL)/p-AlGaN/p-GaN structure. Due to the p-EBL layer and p-AlGaN layer having a higher AlN composition, the Mg ionization efficiency is less than 1% at room temperature, so the free hole concentration can be lower than 10^17^ cm^−3^ [37]. On the one hand, the doping efficiency of the p-type Al-rich AlGaN layer is very low, which seriously affects the hole injection of DUV LEDs. On the other hand, the two energy barriers in the p-EBL/p-AlGaN/p-GaN structure further hinder the hole transport. For this reason, researchers have proposed many new concepts and methods to improve the hole injection efficiency, such as polarization-induced doping, which has been achieved in high hole concentration in AlGaN superlattices and gradient layers [38,39,40]. Secondly, in order to overcome the low efficiency of Mg doping in the p-AlGaN layer, the most commonly used method at present is to use the Mg-delta doping or modulation doping technology [39,41]. According to the report by Chen et al., the hole concentration can be increased to 4.75 × 10^18^ cm^−3^ by using the indium-surfactant-assisted delta doping method [41]. In addition, in the aspect of hole transport in the p-type layer, the barrier height for holes can be suppressed through adopting the p-AlGaN layer with multiple stair-cased AlN compositions or grading the AlN composition [42,43] to improve the hole injection efficiency.

**Figure 1:**
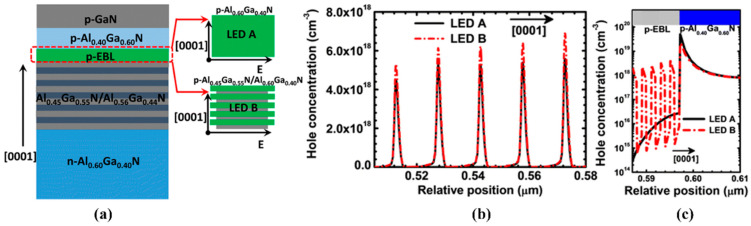
(**a**) Schematic structures for DUV LEDs with p-Al_0.60_Ga_0.40_N EBL (LED A) and p-Al_0.60_Ga_0.40_N/p-Al_0.45_Ga_0.55_N superlattice EBL (LED B). Numerically calculated hole concentration profiles (**b**) in the MQWs, (**c**) in the p-EBLs and p-Al_0.40_Ga_0.60_N layers for LEDs A and B. Reproduced from Ref. [45] with permission from Springer.

Due to the difference in mobility and conductivity, the concentration of electrons and holes is very different, so the electrons in DUV LEDs can easily pass through the MQWs’ active region and leak to the p-side. Therefore, researchers proposed the p-EBL structure to prevent electronic leakage, but this would also reduce the hole injection for the LEDs. At present, many p-EBL structures have been proposed. For example, Zhang et al. proposed an Al_x_Ga_1-x_N/Al_y_Ga_1-y_N (x < y) composite EBL structure to replace the traditional single-layer EBL structure [44]. The IQE of DUV LEDs with a composite EBL structure is higher than that of the traditional single-layer EBL structure, because the composite EBL structure has a higher electron barrier and lower hole injection barrier. In addition, Zhang et al. proposed a p-AlGaN/p-AlGaN superlattice EBL structure for DUV LEDs, where the nearly efficiency-droop-free DUV LED structure can be obtained experimentally [45]. As shown in Figure 1, this structure causes the p-EBL structure to have a high hole concentration, which, correspondingly, improves the hole injection efficiency in multiple quantum wells (MQWs). The next year, an EBL structure with a linear increase in AlN components in the direction of (0001) was proposed, and the experimental observations were further verified through numerical calculations [46]. Compared with the traditional EBL structure, it could generate a polarization-induced electric field, thus, achieving an efficient hole injection.

**Figure 2:**
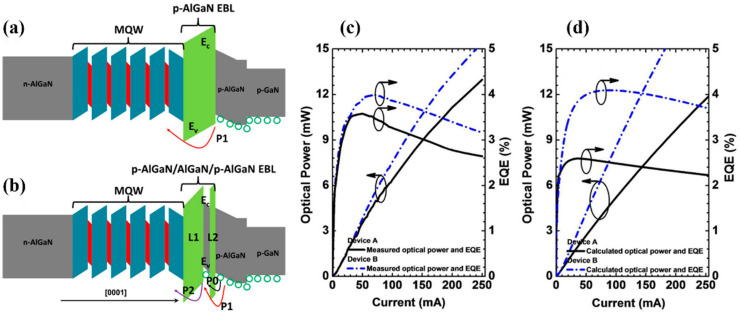
Schematic energy band diagrams for (**a**) DUV LEDs with the conventional p-AlGaN EBL and (**b**) DUV LEDs with the p-AlGaN/AlGaN/p-AlGaN EBL. (**c**) Experimentally tested and (**d**) numerically computed EQE and optical power at different injection current levels for devices A and B, respectively. Reproduced from Ref. [48] with permission from the American Chemical Society.

In addition to some of the methods mentioned above, reducing the thickness of the p-EBL layer can also enhance the hole intraband tunneling efficiency, thus, improving the hole injection [47]. In general, thick p-EBLs result in the intraband tunneling for holes being more difficult. According to a report by Zhang et al., through inserting a very thin layer of AlGaN into a p-EBL, the thermionic emission and intraband tunneling process can be obtained simultaneously in the hole transmission process, reducing the valence band barrier for layer L1 (the p-EBL layer near the side of the quantum barrier), thus, being beneficial for improving the hole injection efficiency [48]. As shown in Figure 2, the experimental and calculated results showed the same trend. Recently, Liu et al. studied the hole injection at the last quantum barrier (LQB) and p-EBL interface. By inserting an Al-composition-increasing AlGaN layer (ACI-AlGaN) between the LQB and p-EBL, the effective barrier height of holes could be reduced, and the hole accumulation could be induced in the vicinity of the n-side interface of the p-EBL, so as to promote hole injection and improve the EQE and optical power of the DUV LEDs [49].

**Figure 3:**
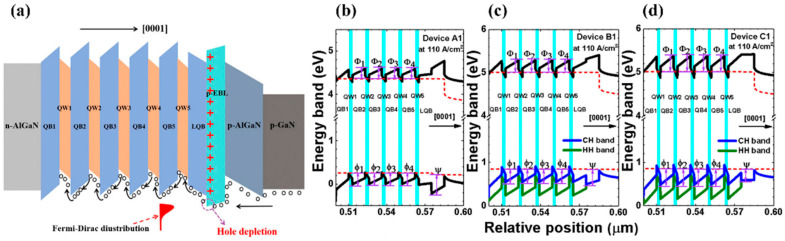
(**a**) Schematic energy band diagram for investigated DUV LED structures which were grown along the [0001] orientation. Polarization-induced positive charges were generated at the LQB/p-EBL interface. Energy band diagrams in the vicinity of the active region, the p-EBL and the partial p-AlGaN layer for (**b**) device A1, (**c**) device B1 and (**d**) device C1 at an injection current density of 110 A/cm^2^. Reproduced from Ref. [50] with permission from AIP Publishing.

In addition, the hole injection efficiency is also related to the composition of the quantum barrier. Zhang et al. effectively improved the hole injection from the p-EBL to the active region of AlGaN-based DUV LEDs by properly increasing the AlN component of the AlGaN quantum barrier and taking advantage of the polarization effect [50]. As shown in Figure 3, the gradual increase in AlN components of devices A1, B1 and C1 led to a decrease in the positive polarization charge density at the LQB/p-EBL interface, thus, inhibiting the hole depletion effect and reducing the effective valence band barrier height of the p-EBLs. Therefore, the hole injection was effectively enhanced and the device performance of the DUV LEDs was improved. The experimental measurements for the EQE and optical power of device A1 were shown to be consistent with the calculated results, which also confirmed the effectiveness of the model.

According to the reports of different research groups, the hole concentration distribution at different positions in the quantum wells for DUV LEDs is different [42,45,48,51,52,53,54,55]. Some of these reports showed that holes were usually clustered in the last quantum well nearest to the p-EBL [45,48,51,53]. In addition, the energy band bias ratio between the different conduction band biases and valence band biases would seriously affect the hole transport in the active region. The energy band bias ratio often mentioned in some reports included 50/50 [45,48,51], 65/35 [42,52] and 70/30 [53,54]. Compared with InGaN/GaN-based visible LEDs, the hole distribution in the active region of AlGaN-based DUV LEDs was shown to be more uniform [45,48,51]. Therefore, we should focus on improving the hole concentration level in each quantum well, rather than studying the hole concentration distribution uniformity in the active region for DUV LEDs [56].

### 2.3. Improve the Electron Injection Efficiency

**Figure 4:**
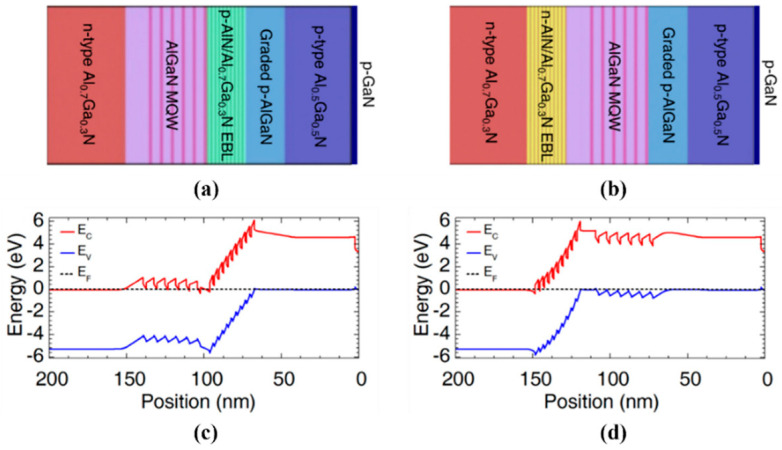
Schematic of the DUV LEDs with (**a**) p-AlN/AlGaN superlattice EBL and (**b**) n-AlN/AlGaN superlattice EBL. Energy band diagram for the DUV LEDs with (**c**) p-AlN/AlGaN superlattice EBL and (**d**) n-AlN/AlGaN superlattice EBL. Reproduced from Ref. [64] with permission from the Optical Society of America.

In addition to improving the hole injection efficiency mentioned above, it is also necessary to research the electron injection and to improve the IQE of AlGaN-based DUV LEDs by enhancing the electron injection. At present, a great deal of research has been conducted on the enhancement of electron injection in InGaN/GaN-based visible LEDs [57,58]. For AlGaN-based DUV LEDs, due to the influence of the unbalanced carrier injection, electrons tend to overflow from the active region. In response to the above problems, researchers have proposed many methods to suppress the electronic leakage of DUV LEDs. The more direct method is to design a p-EBL structure, for example, a p-EBL with an AlGaN insertion layer [48], a p-EBL with a graded AlN composition [59,60], a superlattice p-EBL [45,61,62,63], etc. In addition, some researchers have proposed an n-EBL structure to reduce electronic leakage. As shown in Figure 4, Pandey et al. could effectively reduce the electronic leakage without damaging the hole injection by adding an n-type AlN/AlGaN SL EBL structure before the active region, with experimental measurement results showing that the device performance of the DUV LEDs was significantly improved [64]. In addition to exploring new EBL structures, Liu et al. proposed a new n-type confinement layer with a stepped and superlattice structure [65]. The results showed that the new LEDs with this structure could reduce the effective conduction band barrier height of electrons, thus, facilitating the injection of electrons into the active region and significantly improving the IQE of the DUV LEDs. In addition, researchers also proposed new MQW active region structures to suppress electronic leakage. For example, Sun’s group proposed an Al component gradient quantum barrier (QBs) structure [66] and the stepped QB structure [67], which suppressed electronic leakage and, thus, improved the IQE of the DUV LEDs.

**Figure 5:**
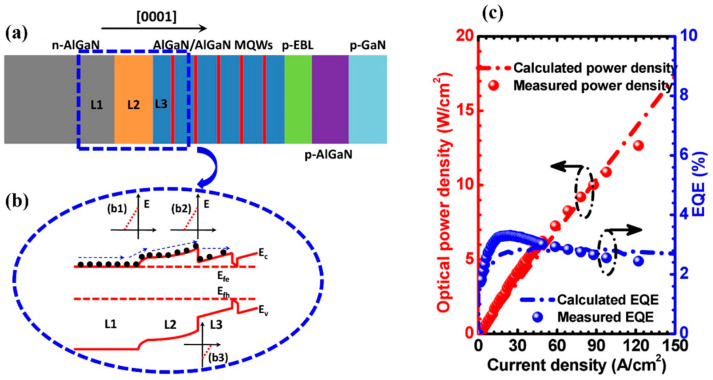
(**a**) Schematic structure for the (0001) oriented DUV LEDs and (**b**) schematic energy band diagram when the electron concentration and the alloy in the n-AlGaN layer were modulated. (**c**) Experimentally measured and numerically calculated optical power density and EQE as the function of the injection current density for device 1. Reproduced from Ref. [69] with permission from the Optical Society of America.

**Figure 6:**
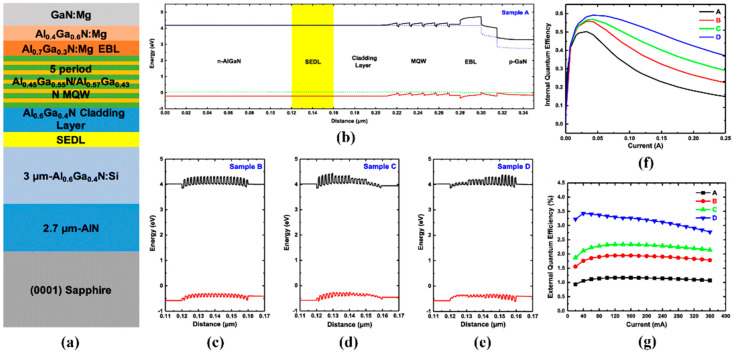
(**a**) Schematic of DUV LEDs. (**b**) Schematic band diagram of conventional DUV LEDs. (**c**–**e**) Three different superlattice electron deceleration layers. (**f**) IQE and (**g**) EQE as functions of current for the four DUV LEDs. Reproduced from Ref. [70] with permission from Springer.

In addition, the electron injection efficiency can also be improved by adjusting the electron drift speed and electron energy of DUV LEDs. According to a report by Zhang et al., the internal electric field can be generated at the n-AlGaN/first quantum barrier interface by adjusting the Si doping concentration of the n-AlGaN layer, thus, reducing the electron drift speed and the electron energy [68]. When the electron drift speed is slow, the MQWs can have more opportunities to capture the electrons. In addition, the polarization-induced electric field can be obtained by gradually reducing the AlN composition of the n-AlGaN layer to “cool down” the electrons, as shown in Figure 5, with experimental measurement values having been well reproduced numerically [69]. The research shows that when the electron energy is reduced to a certain level, the electron concentration in MQWs can increase, thus, improving the device efficiency of DUV LEDs. On the one hand, different doping concentrations in the n-AlGaN layer can cause interface loss; on the other hand, the interface of Al_x_Ga_1-x_N/Al_y_Ga_1-y_N (x > y) can generate a polarization-induced electric field, both of which help to reduce the kinetic energy of electrons, improving their capture. Hu et al. also proposed a new type of DUV LEDs with a superlattice electron deceleration layer (SEDL) structure, which can effectively slow down the electrons injected into the active region and improve the radiation recombination efficiency [70]. The effects of several chirped SEDLs on the performance of DUV LEDs have been investigated experimentally and numerically. As shown in Figure 6, compared with traditional DUV LEDs, DUV LEDs with a SEDL structure had a higher IQE and EQE.

In addition, many researchers have analyzed and discussed N-polar structures. Compared with Ga-polar devices, N-polar LEDs exhibit superiority and potential in suppressing electron leakages and enhancing the carrier injection efficiency. Currently, the advantages of N-polar structures have been proven to inhibit the efficiency droop in visible InGaN-based LEDs. Although the performance of N-polar LEDs is not as good as that of Ga-polar LEDs, it has been demonstrated that the characteristics of N-polar LEDs may be enhanced through structural optimization [71]. Unlike N-polar InGaN visible LEDs, N-polar AlGaN UV LEDs grown at high temperatures are expected to have better performance than Ga-polar devices. Studies have shown N-polar devices to exhibit enhanced carrier injection in both simulations and experiments [72,73]. By studying the performance of N-polar AlGaN-based UV LEDs with different Al contents in quantum wells and barriers, Zhuang et al. found that N-polar structures can suppress electron leakage, effectively improving the IQE and suppressing an efficiency droop, especially for DUV LEDs [74]. Therefore, N-polar structures have great potential in achieving high-efficiency DUV LEDs.

## 3. Increase the Light Extraction Efficiency of DUV LEDs

In general, the EQE of DUV LEDs can be expressed as the product of internal quantum efficiency (IQE) and light extraction efficiency (LEE); the IQE is the ratio of the electron–hole pairs that participate in radiation recombination to the electron–hole pairs injected into the LED device, while the LEE is the ratio of the number of photons emitted into the free space to the number of photons emitted from the active area. In a previous article, some methods for improving the IQE were discussed. In this section, we mainly focused on how to improve the light extraction efficiency (LEE) of DUV LEDs. In general, the LEE of DUV LEDs is generally low, which is caused by their unique optical polarization and optical absorption. At present, the light extraction capabilities of DUV LEDs face many challenges, such as the absorption of deep ultraviolet light by the p-GaN layer and electrodes, total internal reflection (TIR) caused by the high refractive index difference in the interface between the semiconductor and air and the optical emission dominated by TM-polarized photons. In this regard, researchers have proposed various methods to improve the LEE of DUV LEDs, such as growing DUV LEDs on nanopatterned sapphire substrates [75,76,77], using mesh p-type contact electrodes [78,79], roughening the DUV LEDs’ surface [80,81], fabricating inclined sidewalls [82,83,84] and utilizing advanced metal reflectors [85,86,87]. Among them, the design of inclined sidewalls has shown great potential in improving the LEE, because most TM-polarized light tends to escape from the sidewalls of DUV LEDs.

**Figure 7:**
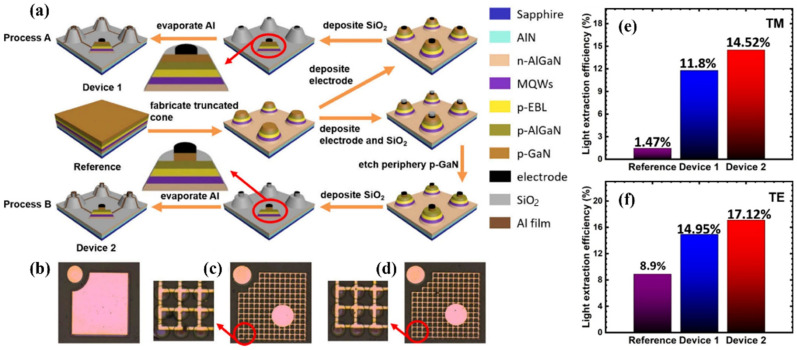
(**a**) Process flow charts for DUV LEDs with inclined sidewalls (process A) and the proposed inclined sidewalls with laterally overetched p-GaN layer (process B). (**b**–**d**) Top views for reference: device 1 and device 2 under optical microscope. LEEs for (**e**) TM- and (**f**) TE-polarized light for reference device: devices 1 and 2. Reproduced from Ref. [92] with permission from the Optical Society of America.

Different research groups have proposed different methods to solve the low LEE problem of DUV LEDs caused by the absorption of deep ultraviolet light by the p-GaN layer and electrodes. Takano et al. for the first time reported that they replaced the traditional p-GaN contact layer with a p-AlGaN to reduce the absorption of light when it propagated upward, with Rh being used as the reflecting p electrode. Through the combination of the two structures, the output power and EQE of the DUV LEDs were significantly improved [88]. In addition to Rh metal, many research groups have also studied other metals with high ultraviolet reflectance, such as Al, Pd, Mg, etc. The RIKEN research group significantly improved the LEE of DUV LEDs by introducing p-AlGaN with high transparency and a Ni/Mg structure p electrode with high reflection [89]. In addition, the structure of highly reflective ultraviolet n electrodes is also a direction worthy of study. Gao et al. reported that the LEE of DUV LEDs was increased by 25.4% when using a highly reflective n-type electrode composed of Cr/Al [90]. At present, the strong optical absorption of the p-GaN layer is still an important limiting factor in enhancing the LEE. For this reason, Fayisa et al. proposed to remove the central region of the p-GaN layer to form a p-GaN ring, thus, improving the LEE of the DUV LEDs [91]. However, the above measures reduced the ohmic contact area and increased the series resistance. Therefore, Zhang et al. proposed a truncated structure with a laterally overetched p-GaN layer; that is, the peripheral p-GaN layer on the truncated base was partially etched [92], as shown in Figure 7. This concept of reducing the area of the p-GaN layer could suppress the optical absorption without increasing the forward bias, thus, effectively improving the LEE of DUV LEDs without sacrificing the electrical performance.

For DUV LEDs, due to large differences in the refractive index, a serious total internal reflection (TIR) can occur at the substrate/air interface and the epitaxial layer/substrate interface. Most photons cannot enter the escape cone, and are absorbed by the material after multiple internal reflections, finally being converted into heat [93]. Therefore, the TIR that destroys the interface is one effective way of improving the device performance of DUV LEDs. In general, researchers roughen the substrate surface to increase the amount of scattering at the substrate/air interface allowing for more photons to escape, thus, improving the LEE of the DUV LEDs. According to the report by Pernot et al., the LEE of 270 nm LEDs was increased 1.5 times by fabricating a moth-eye structure on the back side of a sapphire substrate [94]. Inoue et al. obtained 150 mW high-power 265 nm DUV LEDs by using a large-area AlN nanophotonic light-extraction structure, with an output power approximately 20 times higher than that of traditional flat-surface DUV LEDs [95]. Liang et al. proposed to use nanolens arrays (NLAs) fabricated with nanolithography and a wet-etching technique to improve the LEE of DUV LEDs. The experimental results showed that when using optimized NALs with a radius of 350 nm, the LEE was enhanced by 24.7% [96].

As mentioned earlier, the optical emission of DUV LEDs is mainly dominated by TM-polarized photons. However, for AlGaN-based DUV LEDs, there are two polarization modes of TE and TM light emission, so the LEE of TE-mode light and TM-mode light need to be enhanced. In this regard, different research groups have also proposed various methods for increasing the LEE of the two modes of light. For example, Lee et al. proposed a bottom-emitting sidewall-emission-enhanced (SEE) DUV LED with multiple mesa stripes and inclined sidewalls covered with a MgF_2_/Al omnidirectional mirror, which could effectively improve the LEE of TM-polarized light [97]. Chen et al. proposed and optimized the angle of the sidewalls for the mesa. The results showed that, compared with traditional devices, the DUV LEDs with the best angle of 37.83° produced a 48% optical power enhancement at the injection current density of 35 A/cm^2^, which was attributed to the LEE enhancement of TM-polarized light [82]. In addition, Xing’s research group has conducted a lot of research on realizing DUV luminescence dominated by TE-polarized light in ultrathin (Al) GaN/AlGaN multiple quantum wells or GaN/AlN quantum dot heterostructures, accomplishing various achievements [98,99]. Recently, Zhang et al. studied different scattering mechanisms of nanopatterned sapphire substrates on DUV LEDs TM- and TE-polarized light [100]. The results showed that the NPSS structure could suppress the LEE of TE-polarized light, and that the optical absorption of the p-GaN layer severely limited the scattering efficiency of the NPSS structure. Therefore, the LEEs of TM-polarized light and TE-polarized light were effectively improved by adopting DUV LEDs combined with a NPSS structure and mesh p-GaN structure.

**Figure 8:**
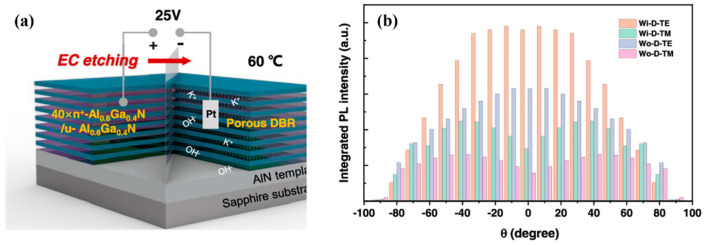
(**a**) Schematic diagram for the structure and fabrication of AlGaN-based NP-DBRs. (**b**) Full spatial TE/TM mode light intensity distributions in the Wi-D and Wo-D regions. Reproduced from Ref. [107] with permission from the American Chemical Society.

In addition to some of the challenges and methods mentioned above, there are many more common strategies for improving the LEE. In practical applications, the reliability and lifetime of LED devices may be limited by the performance of encapsulated materials. As a key characteristic of LED-encapsulated materials, a high refractive index can effectively reduce the total reflection at the interface of LED chips, thereby improving the LEE of the LEDs. Therefore, many researchers have proposed the experimental testing of various encapsulated materials. For example, Kim et al. fabricated a transparent and thermally stable phenyl hybrimer with a high refractive index for LED encapsulation through a siloxane network, the phenyl hybrimer having a high refractive index of approximately 1.56 [101]. Tong et al. used the sol–gel condensation method to synthesize high-refractive-index adamantane-based phenyl epoxy–silicone (APES) resins for LED encapsulation, with APES resins showing a relatively high refractive index of 1.56 [102]. In addition, some reflective structures have also been used to improve the LEE, such as photonic crystals [103], omnidirectional reflectors (ODRs) [104] and distributed Bragg reflectors (DBRs) [105]. Among them, Zhang et al. further researched an ODR’s reflective structure and proposed an evolved reflective system, the full-spatial omnidirectional reflector (FSODR) system, in which all front faces are coated with Al metal [106]. Compared to DUV LEDs without a FSODR, the LEE of DUV LEDs with a FSODR increased by 60%. Recently, Shan et al. researched wafer-scale AlGaN-based deep ultraviolet (DUV) nanoporous (NP) distributed Bragg reflectors (DBRs), as shown in Figure 8; the experimental results showed that due to the reflection effect caused by the NP-DBRs, the light extraction of both the TE and TM modes improved [107].

However, at present, the LEE of AlGaN-based DUV LEDs is still low, resulting in serious reliability problems. Therefore, many methods for the encapsulation of AlGaN-based DUV LEDs have been proposed to improve the LEE and reliability. For example, Ichikawa et al. used direct bonding to fabricate high-output-power DUV LEDs, significantly improving the LEE [108]. Peng et al. proposed a novel packaging method based on an AlN-doped fluoropolymer encapsulation layer, and the results showed that the AlN-doped fluoropolymer structure was more reliable than the silicon and pure fluoropolymer structures [109]. In addition, a liquid packaging structure suitable for DUV LEDs has been proposed, with the reliability test has showing that the liquid packaging DUV LEDs had good thermal and DUV resistance [110].

## 4. Increase the Wall-Plug Efficiency of DUV LEDs

As one of the important parameters for judging the photoelectric performance of DUV LEDs, the WPE can usually be expressed as a product of the external quantum efficiency (EQE) and electrical efficiency (EE); the EQE is the ratio of the number of photons that escape in the air from LED devices to the number of carriers injected into the LED devices, while the EE is the electrical efficiency caused by the voltage loss when the electrode metal contacts the semiconductor layer. Some solutions for improving the IQE and LEE were discussed above. The following focused on how to improve the WPE of DUV LEDs. As we all know, good thermal management is crucial in improving the WPE and output optical power of DUV LEDs. The large amount of joule heat generated by the device can seriously reduce the efficiency, life and output optical power of DUV LEDs. Therefore, the WPE of DUV LEDs can be improved by reducing the joule heat or thermal resistance of the device. For example, Zhang et al. proposed a new honeycomb hole-shaped electrode (HHSE) structure [111]. Compared with traditional interdigital electrodes, the effective current expansion area of DUV LEDs with the HHSE structure increased by approximately 35.3%. The results showed that when the injection current was 280 mA, the WPE of the DUV LEDs with the new electrode structure increases by 28%. Chen et al. studied the effect of different electrode patterns on the photoelectric and thermal properties of DUV LEDs. The results showed that the design of n-type electrodes around the active area was better. Compared with the p-surrounding electrode LEDs, the light output power of DUV LEDs with n-surrounding electrodes increased by 41% [112].

**Figure 9:**
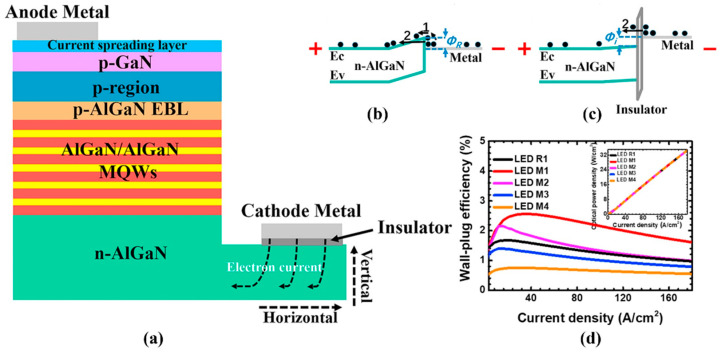
(**a**) Schematic diagram for the proposed MIS-structured DUV LEDs; (**b**) schematic energy band diagram of the n-type metal/semiconductor contact for reference LED; (**c**) schematic energy band diagrams of the MIS structure for the proposed DUV LEDs and (**d**) WPE in terms of the injection current density for LEDs M1–M4 and R1. Inset in (**d**) shows the optical power density in terms of the injection current density for LEDs M1–M4 and R1. Processes 1 and 2 denote the electron transport processes of thermionic emission and intraband tunneling, respectively. E_C_ and E_V_ represent the conduction band and the valence band, respectively. Φ_R_ and Φ_i_ stand for the barrier height for reference LED and different MIS-structured LEDs, respectively. Reproduced from Ref. [113] with permission from Elsevier.

In addition, Shao et al. improved the WPE of AlGaN-based DUV LEDs and reduced the contact resistance by adopting the metal–insulator–semiconductor (MIS) structure on the n-AlGaN layer [113]. As shown in Figure 9, the MIS structure can make the electron affinity band of a cathode metal higher than the conduction band of the n-AlGaN layer, which is conducive to the intraband tunneling effect of electrons. At the same time, the effects of the relative dielectric constant, the band gap, the affinity, the length and the thickness for the insulator, as well as the metal electron affinity on the electronic transport and the WPE of DUV LEDs were also studied. The results showed that using the MIS structure could significantly reduce the influence of cathode metal electron affinity on the WPE. If the relative dielectric constant of the insulator decreased, the WPE of DUV LEDs would increase.

## 5. Conclusions and Outlook

To sum up, this work reviewed some problems and solutions concerning AlGaN-based DUV LEDs. First of all, the IQE is an important factor that affects the EQE of DUV LED devices. On the one hand, due to the lattice mismatch and thermal expansion coefficient mismatch between the AlGaN material and sapphire substrates, the TDD of DUV LEDs is too high, which seriously reduces the IQE. This can be reduced by growing DUV LEDs on a nanopatterned substrate. On the other hand, the IQE of the DUV LEDs is also closely related to the carrier injection. Therefore, in order to improve the EQE of DUV LEDs, we must also enhance the hole injection and reduce electronic leakage. An enhanced hole injection can be achieved by reducing the hole-blocking effect of p-EBL and increasing the hole concentration in MQWs. The reduction in electron leakage can be achieved by designing a new EBL structure, improving the active region structure of MQWs and adjusting the electron drift speed and electron energy. Secondly, the LEE is another important factor that affects the EQE of DUV LED devices. Due to the unique optical polarization and optical absorption, the LEE of DUV LEDs is generally low, but can be improved by optimizing the p-GaN layer and electrode structure, roughening the substrate surface and using the inclined mesa sidewalls. In addition, there are several other well-known strategies for improving the LEE, such as high-refractive-index-encapsulated materials, photonic crystals, ODRs and DBRs. Finally, it was pointed out that the WPE of DUV LEDs can be enhanced by adopting new electrode structures and the MIS structure on the n-AlGaN layer. At present, improving the luminous efficiency and reliability of AlGaN-based DUV LEDs is still a major challenge. Some of the above solutions have solved the device efficiency problem to a certain extent. The key to enhancing the reliability lies in AlGaN-based DUV LED packaging technologies, including direct bonding technologies, fluoropolymer packaging and liquid packaging technologies, improving the LEE and enhancing the reliability. In the future, the IQE, LEE and WPE are expected to introduce a series of cumulative breakthroughs. DUV LEDs are expected to have the same device efficiency and output power as InGaN-based blue LEDs in the near future.
